# Cytotoxic CD4 T cells in the mucosa and in cancer

**DOI:** 10.3389/fimmu.2023.1233261

**Published:** 2023-08-16

**Authors:** Hrishi Venkatesh, Sean I. Tracy, Michael A. Farrar

**Affiliations:** ^1^ Center for Immunology, Masonic Cancer Center, Minneapolis, MN, United States; ^2^ University of Minnesota, Department of Laboratory Medicine and Pathology, Minneapolis, MN, United States; ^3^ Division of Hematology, Oncology and Transplantation, Department of Medicine, University of Minnesota, Minneapolis, MN, United States

**Keywords:** cytotoxic, Tr1 cell, tolerance, stem cell, immunotherapy

## Abstract

CD4 T cells were initially described as helper cells that promote either the cellular immune response (Th1 cells) or the humoral immune response (Th2 cells). Since then, a plethora of functionally distinct helper and regulatory CD4 T cell subsets have been described. CD4 T cells with cytotoxic function were first described in the setting of viral infections and autoimmunity, and more recently in cancer and gut dysbiosis. Regulatory CD4 T cell subsets such as Tregs and T-regulatory type 1 (Tr1) cells have also been shown to have cytotoxic potential. Indeed, Tr1 cells have been shown to be important for maintenance of stem cell niches in the bone marrow and the gut. This review will provide an overview of cytotoxic CD4 T cell development, and discuss the role of inflammatory and Tr1-like cytotoxic CD4 T cells in maintenance of intestinal stem cells and in anti-cancer immune responses.

## Historical overview

Naïve CD4 T cells upon activation differentiate into various specialized subsets that direct specific types of immune responses by producing specific cytokines. In 1986, Mossman and Coffman characterized two main populations of CD4 T cells in mice: IFNγ producing Th1 cells and IL4-producing Th2 cells (1). Subsequently, distinct subsets of CD4 T cells have been described including follicular-helper Tfh cells, IL17-producing Th17 cells, and FOXP3+ regulatory T cells (2–4) among others. Each CD4 T cell subset facilitates immune responses towards distinct types of pathogens through distinct mechanisms.

CD4 T cells have been traditionally associated with helper functions while CD8 T cells have been associated with cytotoxic functions. However, studies as early as the 1970s had identified CD4 T cells with cytotoxic activity in the context of allograft rejection (5–7). Even though subsequent studies identified CD4 T cell clones with cytotoxic potential in humans (8) and mice (9, 10), cytotoxic activity in CD4 T cells was considered an artifact of long-term *in-vitro* cultures. However, recent studies have identified antigen-specific cytotoxic CD4 T cells in patients with chronic viral infections such as CMV (11, 12), HIV (13, 14) and hepatitis (15), and in murine models of chronic viral infection (16). Cytotoxic CD4 T cells have also been shown to be induced in acute influenza infections (17, 18). While the earliest studies identified cytotoxic CD4 T cells in viral infection models, they have also been recently identified in anti-tumor immune responses and in chronic inflammatory responses during auto-immune/auto-inflammatory diseases (19, 20). These studies clearly indicate that cytotoxic CD4 T cells can be elicited under diverse acute and chronic inflammatory conditions *in-vivo*.

Although cytotoxic CD4 T cells were first thought to be a distinct CD4 T cell subset, they have now been shown to have features of various helper T cell subsets. Furthermore, inhibitory CD4 T cell subsets such as FOXP3+ Tregs and FOXP3- Tr1 cells have also been shown to have cytotoxic potential (21, 22). In this review, we summarize the key mechanisms by which CD4 T cells mediate cytotoxic functions, and propose 2 pathways that could regulate the differentiation of cytotoxic CD4 T cells. We also illuminate the role of cytotoxic CD4s in maintaining homeostasis in the gut mucosa and in immune responses against cancer. There will be a special focus on studies that have highlighted novel roles for Tr1-like cytotoxic CD4s with suppressive attributes in maintenance of intestinal stem cells and in cancers of the gut mucosa.

## Identity and differentiation of cytotoxic CD4s

### Mechanisms of cytotoxicity

The fusion of lysosomes to the membrane causes surface expression of the lysosomal proteins LAMP-1 (CD107a) and LAMP-2 (CD107b) - these are markers of degranulation and can be used to identify activated cytotoxic CD4 T cells upon *in-vitro* stimulation. More recently, another lysosomal granule protein NKG7 has been identified as a marker of degranulation of cytotoxic CD4 T cells (23). This has led to the development of a NKG7-Cre transgenic mouse, which when crossed to *Rosa26- LoxP-STOP-LoxP* fluorescent reporter mice can be used to identify cytotoxic CD4 T cells (23). Thus, cytotoxic CD4 T cells can be identified via a variety of markers.

Transcriptomic and proteomic data has shown that cytotoxic CD4s express various granzymes, including Granzyme A, B and K, as well as the cationic antibacterial protein granulysin (24, 25). GZMK and GZMA are also upregulated in Chimeric Antigen receptor (CAR) CD4 T cells, while GZMB and GZMM were enriched in CAR CD8 T cells in CLL (26). Thus, cytotoxic CD4 T cells can mediate killing through granules with cytotoxic function such as GZMB, but also through granules with both cytotoxic and non-cytotoxic functions such as GZMK. Interestingly GZMB+ and GZMK+ CD4s seem to be a part of distinct populations of cytotoxic CD4 T cells in human cancer patients (19). The differentiation pathway and lineage relationship between the GZMB+ and the GZMK+ cytotoxic CD4 T cells are still unknown.

Cytotoxic CD4 T cells are also capable of mediating killing via granule-independent mechanisms using death-inducing receptors. All helper T cell subsets have been shown to have some FasL-dependent cytolytic activity (27). In melanoma cells resistant to FAS-induced apoptosis, the death receptor TRAIL facilitated CD4 T cell-induced cancer cell death (28). The activating NK cell receptor NKG2D has been recently shown to be essential for cytotoxic CD4 T cell-mediated lysis of Tregs (29), thus raising the possibility that NKG2D+ CD4 T cells can mediate MHCII-independent “bystander” cytotoxicity. Signaling via SLAMF7, another NK cell receptor, has also been shown to promote cytolytic activity in CD4 T cells (24). Thus, cytotoxic CD4 T cells employ a number of mechanisms to mediate cell death.

### Transcriptional regulation of cytotoxic molecules in CD4 T cells

Many key regulators of cytotoxic function in CD8 T cells have been shown to be critical regulators of cytotoxic function in CD4 T cells as well. The transcription factor EOMES has been shown to be a key regulator of cytotoxic CD4 T cells in mice and humans via the induction of perforin (30–32). However, other studies show that the transcription factor HOBIT, rather than EOMES, could be essential for expression of cytotoxic molecules in human CD4 T cells (33, 34). Apart from HOBIT, the IL2-dependent induction of the HOBIT homolog BLIMP1 was shown to be critical for induction of granzymes in tumor-specific CD4 T cells (35). Apart from IL2, the cytokine IL15 is also shown to promote the expression of granzyme B in CD4 T cells (35, 36). In CD8 T cells, IL15 is known to induce HOBIT but not BLIMP1 (37). Thus, induction of granzymes in CD4 T cells can be driven by IL2-induced BLIMP1 and IL15-induced HOBIT dependent mechanisms. In CD8 T cells, BLIMP1 is required for induction of Granzyme B in effector CD8 T cells while HOBIT was required for maintenance of Granzyme B in memory CD8 T cells (38). Whether such a temporal regulation occurs in cytotoxic CD4s is not known. While HOBIT and BLIMP1 are essential for induction of granzyme B in human CD4 T cells, they are not required for induction of perforin (39). RUNX3 is known to induce perforin in CD8 T cells (40). Thus, RUNX3 rather than EOMES could be important for inducing perforin in human CD4 T cells. To summarize, EOMES, RUNX3, BLIMP1 and HOBIT have been shown to be key transcription factors that are important for inducing cytotoxic function in CD4 T cells.

### Runx3-dependent and Runx3-independent differentiation of cytotoxic CD4 T cells

**Figure 1:**
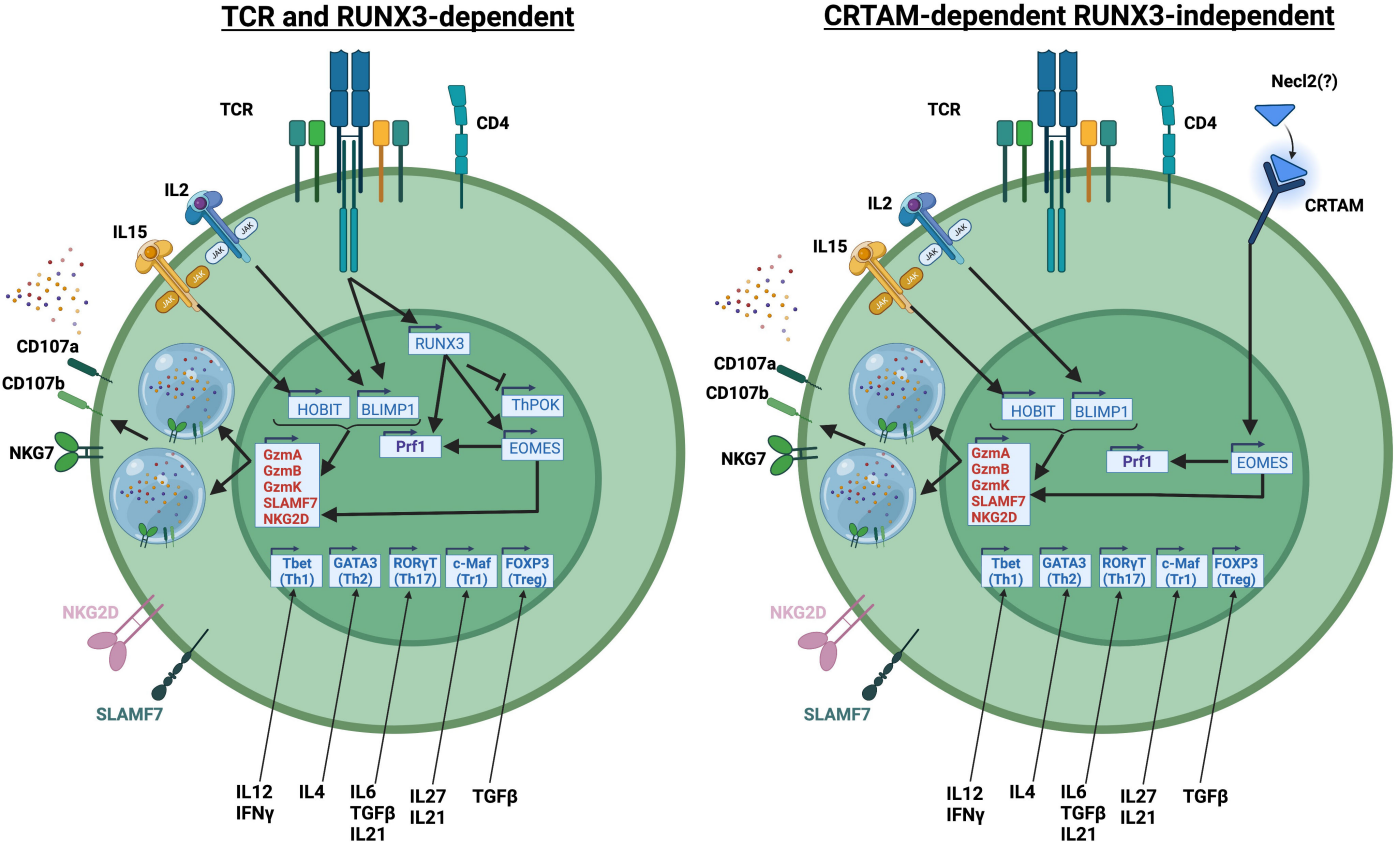
Representation of RUNX3-dependent and RUNX3-independent differentiation pathways of cytotoxic CD4 T cells. Created with BioRender.com.

While the above studies have defined transcription factors that regulate cytotoxic programs in CD4 T cells, the upstream signals that regulate cytotoxic CD4 T cell differentiation have not been as well explored. Based on the literature, there are 2 different pathways for the differentiation of cytotoxic CD4 T cells: a RUNX3-dependent pathway that relies on TCR signaling as the initiating event and a RUNX3-independent pathway that relies on signaling via the receptor CRTAM as the initiating event (summarized in **Figure 1**).

The RUNX3-induced gene EOMES has a well-established role in inducing cytotoxic function in CD8 T cells. However, since THPOK maintains CD4 T cell identity by repressing CD8 T cell lineage genes (including *Runx3*), induction of *Runx3* and *Eomes* in CD4 T cells would likely require downregulation of THPOK. Indeed, a recent study has shown that a significant population of CD4 T cells in mice have downregulated THPOK and upregulated RUNX3, and that these CD4 T cells eventually acquire cytotoxic function in a stepwise manner (41). However, another study showed that cytotoxic CD4 T cells in humans acquire cytotoxic function by upregulation of RUNX3 without repression of ThPOK (33). However, in that study, siRNA knockdown of THPOK showed that THPOK still negatively regulated the cytotoxic program in CD4 T cells. Whether cytotoxic CD4 T cells in humans can downregulate THPOK to improve their cytotoxic function is not known.

In mice, loss of THPOK and de-repression of RUNX3 were shown to be driven by chronic TCR signaling (41). In agreement with the findings from the mouse study, data from human patients has shown that Cytomegalovirus (CMV), a chronic viral infection, strongly expands cytotoxic CD4 T cells (33). CMV-specific cytotoxic CD4 T cells upregulate PD1, an indicator of active TCR signaling, and have reduced ability to produce the Th1 cytokines IFNγ and TNF (42). Interestingly however, the CMV-specific cytotoxic CD4 T cells do not express other exhaustion-associated markers such as TIM3 and LAG3 and are only minimally-responsive to PD1 blockade, suggesting that the TCR-induced cytotoxic phenotype is distinct from exhaustion (42).

In contrast to the RUNX3-dependent model, studies have shown that CRTAM, a molecule that regulates cytotoxic function in CD8 T cells and NK cells, can also induce cytotoxic CD4 T cells (43). Interestingly in this study, CRTAM directly regulates EOMES expression in a RUNX3-independent manner (43). The previously established roles of BLIMP1 and HOBIT in regulating the expression of granzymes and other cytolytic molecules would be compatible with both CRTAM-dependent and RUNX3-dependent models of cytotoxic CD4 T cell development. The factors downstream of RUNX3 or CRTAM that regulate cytotoxic CD4 T cell differentiation have not been well-described.

Although cytotoxic CD4 T cells are considered a distinct subset from helper CD4 T cells, cytotoxic CD4s can develop under different polarization conditions and have transcriptional signatures/features associated with the polarized CD4 T cell subset, including Th1, Th2, Th17, Tregs, and Tr1 cells (44–48). Whether or not the cytotoxic program can be induced in all helper T cell subsets, or whether cytotoxic CD4 T cells acquire different polarized states after differentiation is still unclear: while some studies show that cytotoxic CD4 T cell differentiation is TBET-dependent and occurs most efficiently under Th1-polarization conditions (33), other studies show that the regulation of the cytotoxic program occurs independent of TBET (35).

### Definition of cytotoxic CD4 T cells: phenotype vs function

A major challenge in defining cytotoxic CD4 T cells is that CD4 T cells that express molecules associated with cytotoxic functions are not necessarily cytotoxic. Indeed, a population of GzmK^hi^ GzmB^lo^ CD8 T cells has been shown to have low cytotoxic potential – rather, these cells are key drivers of inflammation (49). Granzyme B has also been linked with non-cytotoxic functions, including regulating the polarization of Th0 and Th1 vs Th17 CD4 T cells (50). Perforin is important for the formation of pores to facilitate granzyme entry into the target cells. Thus, it is possible that expression of perforin, rather than granzymes, is a more reliable indicator of cytotoxic CD4 T cells. Indeed, deletion of perforin or inhibition of perforin activity has been shown to inhibit cytotoxic activity in CD4 T cells (24, 51). Furthermore memory CD4 T cells in humans express comparable granzyme B, but lower perforin, than memory CD8 T cells (52). This observation would fit with both the RUNX3-dependent and RUNX3-independent models of cytotoxic CD4 T cell differentiation. In both models, induction of EOMES and/or RUNX3, transcription factors that induce perforin, is a key step in the development of cytotoxic CD4 T cells.

As mentioned before, CD4 T cells can mediate cytotoxicity through death receptors such as FASL. However, all helper-T cell subsets have been shown to have some basal cytotoxic potential that is driven by the expression of the death receptor FasL (27). Thus, whether “cytotoxic” CD4 T cells expressing death receptors are a distinct subset from helper CD4 T cells is unclear. For this reason, perforin/granzyme expression and perforin-mediated killing remain the most reliable markers of cytotoxic CD4 T cells.

## Cytotoxic CD4 T-cells at the gut mucosal surface

### Introduction

Gut-associated lymphoid tissue (GALT) must maintain a fine balance between suppressive and pro-inflammatory states. This ensures tolerance towards complex commensal microbial communities while also eliciting a population of antigen-specific memory/effector populations poised for rapid response to pathogens. The GALT must do this while also maintaining barrier integrity and integrating signals that dictate the appropriate differentiation of immune subsets from multipotent progenitors. The complex topography of the GI tract influences the cross-talk between CD4 T cells and surrounding cells, which bidirectionally influences both epithelial and T-cell differentiation. Thus, CD4+ T-cells not only comprise a critical population of effector/helper cells, but additionally serve as a central nexus of GALT tissue signal integration.

Millions of crypt and villus structures comprise the epithelium of the gut. Under the epithelium exists the lamina propria and specialized lymph tissue known as Peyer’s patches. The gut epithelium itself is a single layer which is renewed by intestinal stem cells (ISC) every 3-7 days (53, 54). CD4+ T cells may be found in the lamina propria and Peyer’s patches, as well as the intestinal epithelium. Single-cell studies demonstrate that CD4+ T-cells are imprinted, during transition through the mesenteric lymph nodes, with transcriptional programs unique to either the lamina propria or the gut epithelium, implying differentiation occurs prior to tissue migration (55). Entry of CD4+ T-cells to the epithelium requires activation via interaction with antigen-presenting cells in peripheral sites, principally including Peyer’s patches and draining mesenteric lymph nodes (56–58). This results in upregulation of the homing receptor AlphaE/Beta7 (αE/β7). Migration to the epithelium coincides with an increase in cytotoxic potential. These intraepithelial αE/β7+ CD4s are poised to release effector cytokines IFNγ and TNF. FOXP3+ Tregs are restrained to the lamina propria and must lose FOXP3 in order to migrate into the epithelium (58). Thus, most CD4+ T-cells found in the epithelium are antigen-experienced long-lived resident effector/memory T-cells, poised for rapid activation and cytotoxicity (59).

Cytotoxic potential manifests in several transcriptionally diverse CD4+ T-cells of the gut, elicited under a variety of pathogenic challenges and disease states. These include phenotypically recognizable Th0, Th1, Th2, Th17, and Treg cell types (60). Expression of the cytotoxic markers *Nkg7, FasL*, and *GzmB* has been most closely associated with T-helper type 1 differentiation. Th1 cells are recognized by expression of IFNγ and the transcription factor TBET, but modern single-cell studies now challenge the concept that Th1 cells are a discrete population. scRNAseq analysis of murine colonic LP CD4 T cells instead revealed that CD4 T cells develop along a polarized transcriptional continuum (61). Transcriptional variation across single-cell clusters was determined more by the specific pathogen threat than the expression of cytokines or the master transcription factors TBET, GATA3 or RORγ. For example, while *Salmonella* classically elicits a phenotypic Th1 response, challenge with Salmonella led to the development of several clusters of effector CD4 T cells with varying expression levels of Th1-defining gene sets; *Ifng* and *Tbx21* were diffusely expressed throughout each cluster. Furthermore, single progenitor cells could give rise to cells capable of expressing different canonical cytokines. Similar results were observed for cytotoxicity markers *GzmB*, *FasL*, and *Nkg7*. Caution should therefore be used when describing an association between cytotoxic markers and Th1 cells in the gut, given the broad transcriptional heterogeneity of T-helper subsets observed in this region.

Regardless of their specific co-existing helper functions or cytokine profiles, the development of cytotoxic CD4+ T-cells critically depends on the activity of the transcription factor THPOK. THPOK and RUNX transcription factors are critical for the induction of CD4 and CD8 T-cell differentiation during thymic development. DN thymocytes upregulate RUNX1 as cells transition to the DP stage (62). During subsequent MHCII-induced selection THPOK is up-regulated progressively as thymocytes down-regulate CD8 and become CD4+ SPs. RUNX3 and THPOK mutually antagonize each other during CD4 and CD8 T-cell thymic development, with CD4+ T-cell differentiation dependent on THPOK expression. In the periphery, CD4+ T-cells continue to express THPOK, which represses genes of the CD8 lineage including cytotoxic genes GzmB and *Prf1* (63). A key observation was that mature CD4+ T-cells in the gut could downregulate THPOK in response to strong or chronic TCR activation, leading to re-expression of cytolytic machinery and generation of MHCII-restricted CD4 T-cells (41). THPOK downregulation is dependent on engagement of the TCR-MHCII and PD1/PDL1 axes (64). This likely leads to disruption of a positive-feedback auto-regulatory loop wherein THPOK expression is maintained via binding and repression of a silencer element (*Sil^Thpok^*). Disruption of THPOK expression is dependent on TBET-mediated re-expression of RUNX3, which binds to the *Sil^Thpok^* element (65). Thus, CD4+ T cell cytotoxic potential appears dependent on a TBET-RUNX3-THPOK silencing axis, leading to re-expression of canonical CD8+ cytolytic molecules.

Downregulation of THPOK induces expression of CD8αα, coincident with re-expression of *GzmB* and *Prf1*, leading to the development of double positive (CD4/CD8αα) cytotoxic intraepithelial lymphocytes. These CD4/8 DP IELs have significant anti-inflammatory properties, similar to FOXP3+ T-regulatory cells, and can in fact originate from either CD4+ conventional or FOXP3+ pTreg precursors (58, 66–69). This was shown by multiple groups applying lineage tracing using *Rosa26-LoxP-STOP-tdTomato* reporters, and single-cell techniques, that enable identification of ex-FOXP3+ cells (55, 70). Treg cells (Tomato+ FOXP3+) began in naïve states, then expanded into a Tomato+ FOXP3- subset (ex-Treg, or Treg-like) before differentiating into pre-intraepithelial lymphocyte (Pre-IEL) subsets, and finally a CD4+CD8αα+ intra-epithelial population. Treg-like cells had downregulated Foxp3 and acquired some markers of cytotoxicity (*Nkg7, GzmB)* and chemokine expression (*Ccl5)*. CD4+ T-conventional (Tomato-) cells also transited through naïve and pre-IEL subsets with similar gene expression profiles, before also terminally differentiating into CD4+CD8αα+ cells. TCR diversity diminished as both Treg and Tconv populations differentiated, implying clonal selection. TCR clones were shared between populations comprising the Treg differentiation pathway, and between populations comprising the Tconv differentiation pathway, although not between populations of these two pathways, supporting the independence of the Treg and Tconv lineages. Functional experiments confirmed that TCR engagement by MHCII molecules expressed by epithelial cells was critical for differentiation of CD4 precursors into pre-IEL and CD4+CD8αα stages (64). These THPOK- CD4 T cells appear poised to exert either immunosuppressive or pro-inflammatory functions depending on surrounding inflammatory signals, and complement other suppressive populations. OT-II mice deficient in RUNX3 were unable to downregulate THPOK or develop CD4+ IELs; in response to OVA exposure, they developed colitis. This phenotype could be rescued by provision of THPOK-deficient OT-II CD4 T cells. IL15 exposure led to expression of the cytotoxic marker CD107 on OTII-specific THPOK- CD4+ T-cells, whereas it had no effect on THPOK+ CD4+ T-cells (41). Thus, the majority of cytotoxic CD4+ T-cells of the gut epithelium are CD4+CD8αα DP cells, which originated as either FOXP3+ pTregs or FOXP3- Tregs and developed via interactions with epithelial cells presenting antigens in an MHCII context. Their ultimate functionality depends on the cytokine milieu of the epithelium, with IL15 playing a principal role in cytotoxic capacity.

### T-regulatory type 1 cells

In addition to FOXP3+ T regulatory cells and CD4+CD8αα DPs, a third class of potentially cytotoxic T-cells with regulatory potential has been described. T-regulatory type 1 (Tr1s) were first described as arising in a patient with severe combined immunodeficiency (SCID) who received mismatched allogeneic fetal liver and thymus transplants and developed stable mixed chimerism (71). Subsequent studies have demonstrated the importance of Tr1 cells at preventing allo-rejection in bone marrow and solid organ transplantation settings. Clinical efforts to utilize these properties have demonstrated the ability of Tr1 cell therapy to tolerize patients towards solid organ grafts from HLA-mismatched donors, potentially greatly expanding organ transplant candidacy, and preempting the need for lifelong immunosuppression in graft recipients. Tr1s are FOXP3- IL10-producing CD4 T cells with the potential to suppress immune responses to foreign- and self-antigens. Tr1s are not the only FOXP3- CD4+ cell type that can synthesize IL-10, and current studies do not fully address the lineage relationships between Tr1s and other IL-10-producing FOXP3- CD4 T cells. In settings of chronic infection, Th1 cells are known to switch to high production of both IFNγ and IL10 (72). However, Th1 production of IL10 may be transient, in contrast with the more sustained production observed by Tr1 cells. Tr1s produce relatively lower levels of *Tbx21* and *Egr2*, but elevated levels of *Gata3* as compared to IL10-producing Th1 cells, potentially hinting at separate developmental pathways and enabling their discrimination (73, 74). Phenotypically, Tr1s were proposed to be identifiable by CD49b and LAG3 positivity, in both humans and mice, although several reports of IL10-producing FOXP3- CD4 T cells did not evaluate them for these markers (75). Thus, for the purposes of this review, Tr1 cells are defined as antigen-experienced FOXP3- IL10+ CD4+ T-cells with well-demonstrated roles suppressing inflammation towards self-antigens, particularly in alloreactive settings.

### Development and function of Tr1s

Tr1 cells originate from naïve CD4 T-cells in the periphery following chronic antigen stimulation in the presence of key cytokines including IL10 (76–79). IL10 appears sufficient but dispensable for Tr1 induction, and yet critical for maintenance of Tr1 suppressive function; Using dual-reporter *Foxp3* ^gfp^/IL-10^Thy-1.1^ mice crossed to an IL10-deficient background, Maynard et al. showed that IL10 producing Tr1 cells continued to be found in normal frequencies in the epithelium and lamina propria of both the small and large intestine (80). However, IL10R-impaired CD4+ T-cells have deficiencies in IL10-secretion and are less capable of suppressing colitis (81). This demonstrates that IL10 can function in paracrine and/or autocrine manner to induce and sustain the maximal activity of Tr1 cells and is a key mediator of Tr1 suppressor activity. Other signals important for Tr1 differentiation include IL27 and TGFβ. An emerging model suggests that Tr1 induction requires (i) STAT1/3 activation via IL27 and/or IL10, (ii) aryl hydrocarbon receptor (AHR) or c-Maf upregulation, stimulated via TGFβ or ICOS-L binding, and (iii) TCR stimulation by cognate antigen presented in an MHCII context (reviewed in (82)). IL21 may also contribute, functioning in an autocrine manner to maintain Tr1 cells, as well as having pleiotropic immunomodulatory effects on several surrounding cell types. Overall, Tr1 induction and maintenance appears to depend on antigen stimulation, IL10 or IL27 exposure, and sustained autocrine stimulation via IL10 and/or IL21.

In addition to roles for AHR and c-MAF described above, recent evidence demonstrates the importance of the transcription factor EOMES at mediating Tr1 development, and in particular, at inducing cytotoxic potential among Tr1s [reviewed in (83)]. The majority of evidence to date has not clarified whether Tr1 cells are a distinct differentiation lineage, or simply a transient activation state. Recent evidence however posits that human EOMES+ Tr1s are a unique lineage of GZMK+ CD4+ T-cells (84). As described above, EOMES and TBET have a high degree of homology but have differential expression patterns; TBET is expressed in most CD4+ T-cells early during acute inflammation, and in particular under Th1-polarizing conditions. In contrast, EOMES induction seems to occur only after prolonged inflammation, e.g. >30 days in LCMV mouse models. EOMES, in concert with TBET, induces both *Il10* expression and the cytotoxicity genes *GzmB*, *Nkg7*, *Prf1*; in human Tr1s GzmK is induced via direct binding of TBET and EOMES to its promoter. EOMES has been proposed as a possible lineage-defining factor for Tr1 cells, which is congruent with its capacity to block *Foxp3* expression (32, 84–86). However, EOMES is not specific to Tr1-cells, and not all reported Tr1-like cells express EOMES, confounding efforts to perform lineage tracing based solely on this marker (75). Fate-mapping experiments, ideally using dual-reporter FOXP3/IL10 mice should conclusively reveal the origins of these cells under different contexts.

Tr1s expressing granzymes, perforin, and/or granulysin have been observed to mediate direct cytolysis of myeloid cells. Most studies of Tr1 cytotoxicity have focused on their capacity to induce death of myeloid APCs. Clinical-grade *ex-vivo*-expanded Tr1 cells with efficacy in suppressing Crohn’s disease were found to be cytotoxic towards myeloid cells over a timespan of several days (87). This effect was independent of perforin, but dependent on the activity of granzymes as well as granulysin, an antimicrobial peptide (88). Additionally, it was observed that IL10-producing CD4+ T-cells, expanded *in vitro*, were capable of killing APCs that presented islet autoantigens in an MHCII context, presumably preventing the development of islet autoantigen-specific T-effector cells in a linked-recognition mechanism (89). However, the majority of CD4+ T-cells examined were FOXP3+, suggesting that Tr1s were a minor contributor to this particular effect. A further nuance to many of these studies it that they rely on stimulation with anti-CD3 antibodies, which triggers expression of granzyme B in most T-cells (90). These caveats prompt the question of whether Tr1-mediated cytotoxicity has physiological relevance in unmanipulated *in-vivo* settings. EOMES+ Tr1 cells expressing granzymes and CD107a are overrepresented in patients with chronic lymphocytic leukemia (CLL) and B cell acute lymphoblastic leukemia (B-ALL) (91, 92). Cytotoxic CD4+ T-cells are also found in multiple solid tumor settings, but whether these are predominantly Tr1 cells is not established [reviewed in (19)]. FOXP3+ Tregs are also reportedly cytotoxic, in a perforin- and/or granzyme B-dependent manner (89, 93). Overall, it appears that Tr1s may induce direct cytotoxicity, particularly towards professional antigen-presenting cells, as part of their suppressor and anti-cancer functions. This raises the question as to whether they may also mediate direct cytotoxicity towards alternative antigen-presenting cells such as intestinal epithelial and stem cell populations, as discussed below.

### Tolerogenic functions of T-regulatory type 1 cells vs Tregs in the gut

Recent studies have shed light on previously under-appreciated roles for FOXP3- Tr1 cells, vis-à-vis Foxp3+ Tregs, in maintaining gut homeostasis. Although both Tr1 and Treg cells play important roles in dampening inflammation and mitigating dysbiosis, subtle differences in their anatomic distributions may point to non-overlapping functions. In the lamina propria of mice, Tr1s comprise the major IL10 producing subset of the small intestine, while FOXP3+ Tregs are more common in the large intestine (94). Similarly, chronic stimulation of T-cells in IL10-reporter mice *in vivo* demonstrated predominant induction of Tr1 cells in the small intestine but FOXP3+ Tregs in the colon (95). In mouse models of bone marrow transplant EOMES+ Tr1 cells outnumber FOXP3+ Tregs in the lamina propria by about 4:1, and their presence is critical for the prevention of Graft-versus-host disease (86). Tr1 cells prevent colitis in adoptive transfer models, and pathogenic colitis-inducing Th17 cells are suppressed by both Foxp3+ and Foxp3- IL10-producing CD4+ T-cells (75, 77, 96). Studies of tissues from patients with Inflammatory Bowel Disease (IBD) found that an EOMES+ IL7R- CCR5+ Tr1 population is diminished in the inflamed intestinal lamina propria of patients with IBD, relative to matched healthy control tissue (84). Patients with Inflammatory Bowel Disease are known to have selective loss of IL10 secretion from Tr1 cells, while IL10 production from FOXP3+ Tregs appears unaffected, further emphasizing a specific importance of Tr1s to IBD (97). Importantly, adoptively transferred CD4+ FOXP3- cells are able to convert to FOXP3+ cells in recipient animals, but FOXP3+ cells were not observed to convert into FOXP3- cells, implying that Treg to Tr1 conversion is rare (80). TCR clonotype sharing has been observed between Tr1 and Th1 T cells but not FOXP3+ Tregs in colorectal cancer (98). Finally, peripherally-induced Tregs have a more defined role against foreign antigens, including the commensal bacteria found at higher concentrations in the large bowel, while Tr1s mediate activity against self-antigens. Thus, evidence to date suggests that Tr1 and Foxp3+ T-cells have distinct lineage origins, and rarely interconvert, but serve similar functional roles in distinct anatomic locations. Further research is needed to fully define non-redundant functions between these cell lineages.

### Crosstalk between cytotoxic CD4 subsets and gut epithelial cells, and the potential for cancer initiation

Emerging evidence demonstrates that CD4+ T-cells influence the function and differentiation trajectories of intestinal stem cells (ISCs). ISCs reside in the crypt base and are surrounded by either Paneth cells in the small intestine or deep crypt secretory cells in the colon. They initially develop into progenitors called transient amplifying cells (TA), that proliferate and further differentiate into secretory cells (Paneth, goblet, enteroendocrine, and tuft) and absorptive cell types (M cells, and enterocytes). WNT, NOTCH, and BMP pathways converge to maintain the balance between self-renewal and progressive differentiation of ISCs. Reserve pools of slow-cycling cells reside just above the crypt base, and are able to reconstitute the ISC population after injury (53, 99–103). Additionally, lineage-committed progenitors and terminally differentiated cells may dedifferentiate into stem-like cells, thereby serving as extra “reserve” cells for ISC renewal (104). Overall, the gut epithelium is maintained via a complex milieu of progenitor cell types that can deviate from a hierarchical maturation pattern under conditions of inflammation or injury. At several steps in this process, perturbations in CD4+ T-cell-epithelial interactions may influence cancer initiation and progression.

CD4+ T-cells coordinate ISC differentiation fates that are appropriate to the type of pathogen encounter or inflammatory stimulus. This was showed elegantly by Biton et al, who used single-cell methods to determine that intestinal stem cells are comprised of at least 3 distinct subsets, ISC-I, ISC-II-, and ISC-III (105). Each ISC subset expressed MHCII molecules and presented antigens to CD4+ T-cells. Co-culture with different Th cell types skewed terminal differentiation towards different terminal states; for example, Th1 co-cultured ISCs differentiated towards Paneth and goblet cell lineages, while Th17 cells induced a TA cell fate. IL10 or co-culture with inducible IL10-producing Tregs led to ISC self-renewal and expansion, while co-culture with other Th cytokines downregulated self-renewal in favor of increased differentiation. The provision of IL10 by CD4+ T-cells in the ISC niche is reminiscent of recent independent findings of a similar phenomenon in the hematopoietic stem cell niche (106). It is intriguing to speculate that CD4 provision of IL10 may be a common phenomenon in all stem cell niches. IL10 provision in this niche may maladaptively contribute to early immune evasion by inhibiting the activity of cytotoxic T-cells that could otherwise remove mutation-harboring stem/progenitor cells. While this mechanism requires cognate recognition of MHCII expressing-ISCs, loss of MHCII expression from IECs/ISCs may also increase carcinogenesis. In one study, mice fed a high-fat diet developed alterations in commensal bacterial populations, leading to reduced IFNγ signaling and downregulation of immunomodulatory (*H2-Aa,H2-Ab1,Ciita*) and costimulatory molecules (*Icosl,Sectm1a,Sectm1b*) on ISCs/IECs (107). MHC class II-negative (MHC-II-) ISCs lacking the tumor suppressor APC, coupled with a high fat diet, exhibited greater tumor-initiating capacity than their MHC class II-positive (MHC-II+) counterparts. This suggests that surveillance of MHCII-expressing ISCs/IECs may be an important mechanism of cancer prevention, and suggests a possible mechanistic link between high-fat diets, shifts in commensal bacterial populations, and colon cancer initiation.

Further evidence supporting the importance of CD4+ T-cells in cancer initiation comes from a recent large atlas study of colonic epithelium (108). Mice in which the tumor suppressor APC gene was conditionally deleted in their ISCs (LGF5-CreERt2;ApcL/L) exhibited 2-fold greater tumorigenicity in an orthotopic, syngeneic colon transplantation assay. The greater rate of tumor incidence was not observed when transplanted into mice deficient in B- and T-cells, supporting the role of adaptive immune cells at preventing tumor initiation. In subsequent experiments, APC-mutated colon cancers were then initiated from either stem cells or differentiated cell precursors. Strikingly, despite identical mutational processes, these tumor types developed divergent immune microenvironments. Stem-cell-originating cancer cells co-localized with CD4+ T-cells expressing a high frequency of FOXP3+ cells, as well as upregulation of genes associated with exhaustion including *Pdcd1, Ctla4*, and *Havcr2 (*TIM3). GZMB*+* T-cells were relatively infrequent. Mechanistically, Tregs may constrain cytotoxic CD4+ T-cell development in this context by sequestering excess IL2, thereby preventing BLIMP-1 and GZMB expression (35). In contrast to stem-cell originating cancer cells, tumor cells originating from differentiated cells expressed more robust antigen-presenting machinery and were enriched with a relatively high frequency of cytotoxic CD4+ and CD8+ T-cells. Differences in these mouse tumor models correlate with divergent human precancerous lesions – specifically, serrated polyps and conventional adenomas. Thus, the degree of infiltration by cytotoxic, IFNγ-rich T-cells in early cancer lesions reflects the inherent stemness and antigen-presenting capacity of the tumors cell of origin; these poised cytotoxic T-cells may be an independent variable, aside from tumor mutational burden, that affects clinical responsiveness to checkpoint blockade (109).

A final mechanism by which cytotoxic CD4+ T-cells contribute to tumorigenesis in the gut may be via chronic inflammatory signaling. Regulatory T-cell populations, including Tr1s, are depleted in inflammatory bowel disease (IBD), while inflammatory and cytotoxic CD4+ T-cells are relatively overrepresented (110). Inflammatory cytokines including IFNγ and TNF can damage the Paneth cell niche and T-cell-delivered IFNγ can delete ISCs via activation of JAK1/STAT1-mediated apoptosis (111–113). Recognition of ISCs/IECs by CD4+ T-cells can occur via MHCII-independent mechanisms. For example, in Crohn’s disease a unique population of CD4+ NKG2D+ lamina propria lymphocytes were found to functionally interact with epithelial cells via recognition of the stress protein MICA (114). Congruently, therapeutic blockade of αEβ7^+^ receptors, which is a promising clinical strategy in IBD, leads to greater efflux of cytotoxic/proinflammatory CD8+ and CD4+ T cells from the gut to the draining lymph nodes. This treatment does not affect T-regulatory cells, which express low levels of αEβ7^+^ (115). Additional studies are needed to determine whether strategies which diminish chronic inflammation in the gut lower the risk of colon cancer associated with IBD.

## Role of cytotoxic CD4 T cells in immune responses against cancer

### Importance of cytotoxic CD4 T cells in anti-tumor immune responses

A plethora of studies have described CD8 T cells as being the main T cell subset responsible for killing tumor cells and thus a critical component of anti-tumor immune responses. Within this framework, Th1-like helper CD4 T cells have been shown to be important for effective priming of anti-tumor CD8 T cells while CD4+ regulatory T cells have been shown to impede priming and effector function of anti-tumor CD8 T cells (116, 117). This is true both in the context of self/tumor-associated antigens, and mutation-derived neoantigens (116, 118). Indeed, a lower ratio of Tregs to non-Tregs has been associated with improved prognosis in some, but not all cancers (119). While CD4 T cells have been typically relegated to the ‘supporting cast’ of anti-tumor immune cells, studies in the last decade or so have shown that CD4 T cells with cytolytic properties are capable of being the main tumor-killing cell type in effective anti-tumor immune responses. The earliest evidence of this was studies by Jim Allison and Paul Antony showing that tumor-specific CD4 T cells transferred into lymphopenic hosts developed a cytotoxic phenotype upon tumor challenge (120, 121). These cytotoxic CD4 T cells were sufficient to kill tumor cells in a MHCII-dependent manner.

While the above studies were done in murine models of melanoma, this has also been shown in cancers of the mucosa such as mismatch repair sufficient colorectal cancer. This is a subtype of colorectal cancer that is associated with significantly worse outcomes than mismatch repair-deficient colorectal cancer. A recent study showed that GZMB+ cytotoxic CD4s that infiltrated into the center of the tumor were associated with improved prognosis in patients with MMR-sufficient colorectal cancer (122). Furthermore, the beneficial effects of neoadjuvant chemotherapy were associated with infiltration of cytotoxic CD4 T cells into the tumor center. Similar findings were also shown in patients with bladder cancer, where clonally expanded cytotoxic CD4 T cells were enriched in the tumor vs the surrounding healthy tissue and predicted response to anti-PD1 blockade (123). Interestingly in contrast to CD8 T cells, cytotoxic CD4 T cells were shown to be distinct subset from exhausted CD4 T cells. Apart from primary tumors, tumor-specific cytolytic CD4 T cells have been shown to be important for control of lung tumor metastases (124).

### Cancer-associated cytotoxic CD4s share features with canonical T helper subsets

As mentioned before, cytotoxic CD4 T cells have been shown to have features of different helper T cell subsets - this has also been seen in cancer patients. The presence of cytotoxic CD4 T cells that have shared TCR clones with multiple helper T cell subsets suggests that the cytotoxic function in CD4 T cells is regulated independently of their helper function in cancer. Indeed, a recent study showed that IL2 and BLIMP1 induced cytotoxic function in tumor-specific CD4 T cells, and that this occurred independent of TBET-mediated induction of a Th1 polarization state (35). A recent study has shown that CD4 T cells specific for the cancer-testis antigen NY-ESO express genes related to multiple helper T cell subsets at levels comparable to conventional helper T cells (24). Furthermore, cytotoxic CD4 T cells from patients with breast cancer, liver cancer and head and neck cancer had significant clonal overlap with non-cytotoxic Th1, Th2 and Th17 CD4 T cells (24). Thus, cytotoxic CD4 T cells in cancer have attributes of other helper T cell subsets.

### Pro-tumor and anti-tumor role of cytotoxic FOXP3^−^ regulatory (Tr1) T cells in cancer

**Figure 2:**
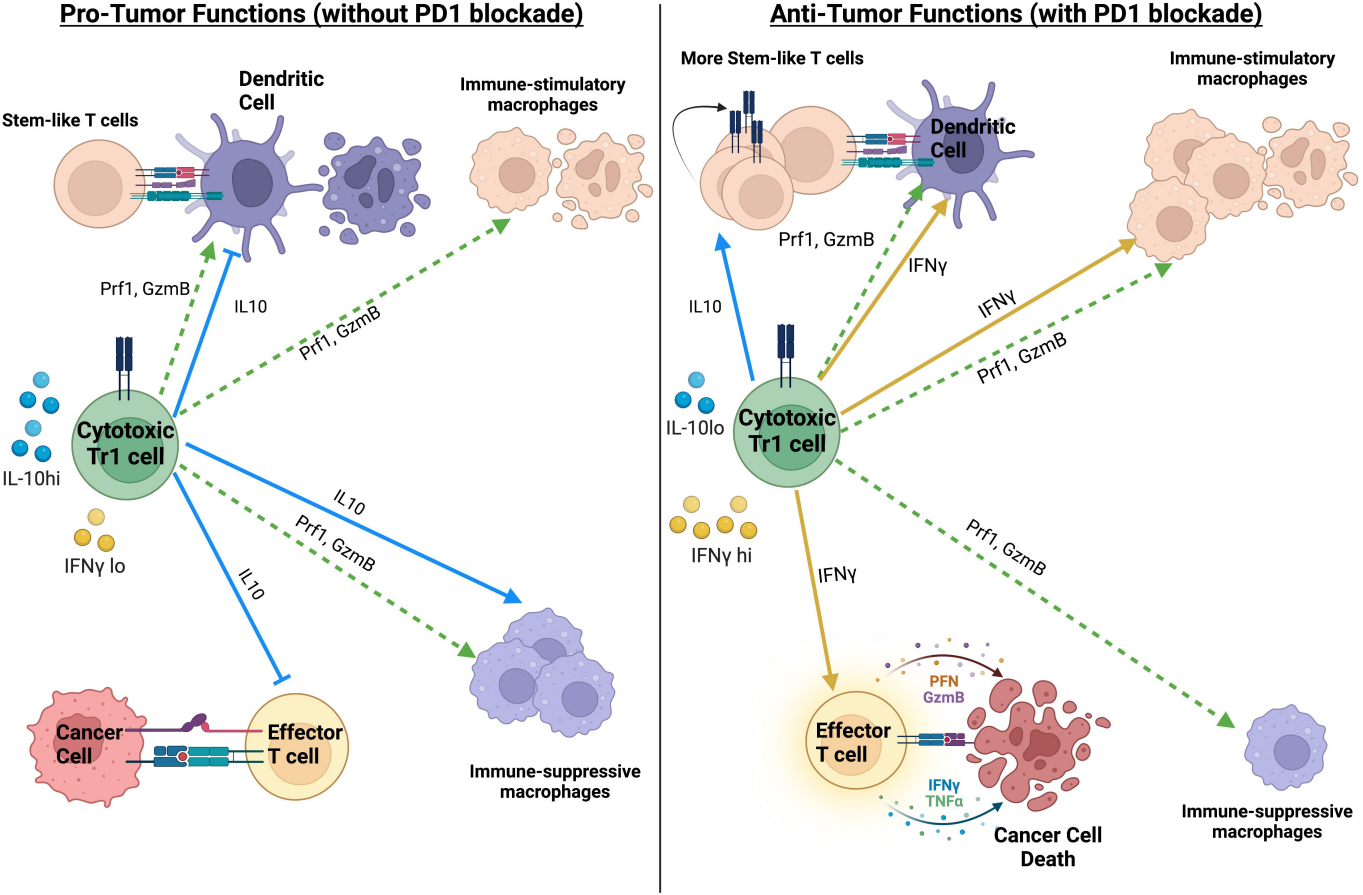
Summary of the pro-tumor and anti-tumor impact of Tr1-like cytotoxic CD4 T cells, in the absence or presence of PD1 blockade. The net impact of Tr1-like cytotoxic CD4 T cells on anti-tumor immunity likely depends on the ratio of IL10 to IFNγ. Created with BioRender.com.

As mentioned before, cytotoxic CD4 T cells are capable of exhibiting attributes of various helper T cell subsets. However, immune-suppressive CD4 T cell subsets have also been shown to have cytotoxic function. These include FOXP3^+^ regulatory T cells (Tregs) and FOXP3^−^ type-1 regulatory (Tr1) cells (47, 48). The role of Tregs, including their cytotoxic function, in anti-tumor immune responses has been extensively discussed in other reviews (125, 126). While FOXP3^−^ type-1 regulatory (Tr1) cells have typically been associated with impairment of anti-tumor immune responses, recent studies have shown that Tr1 cells with cytotoxic function have context-dependent pro-tumor or anti-tumor roles (summarized in **Figure 2**). The most prominent studies highlighting the anti-tumor role for cytotoxic Tr1-like T cells comes from acute myeloid leukemia (AML), where cytotoxic Tr1 cells are known to efficiently kill myeloid leukemia cells while also suppressing Graft-Versus-Host Disease in bone marrow transplant patients (48, 127, 128). Interestingly although the Tr1-cells specifically killed AML blasts, this cytotoxicity was antigen-independent but MHC (HLA)-class I dependent (127). The cytotoxic activity of cytotoxic Tr1 cells against myeloid cells has also been extended to tumor-associated macrophages. Indeed in metastatic melanoma, cytotoxic GZMB^+^ Tr1 cells but not Tregs inhibit macrophage-mediated tumor growth via direct killing of the tumor-promoting macrophages (129). However, the cytotoxic Tr1-cells can also kill antigen-presenting cells that are critical for priming anti-tumor immune responses (130). Indeed, cytotoxic GZMK^+^ EOMES^+^ Tr1 cells have been associated with tumor progression in colorectal cancer, non-small-cell lung cancer, and in liver metastases (91, 98). Whether or not cytotoxic Tr1 cells equally target pro-tumor and anti-tumor myeloid cell subsets is still unclear. Additionally, the immune-suppressive impact of the Tr1-produced cytokine IL10 likely results in a net pro-tumor effect of the cytotoxic Tr1 cells (98). Thus, although cytotoxic Tr1-like cells have pro-tumor and anti-tumor roles, they likely have a net pro-tumor effect in the absence of immunotherapy.

### Importance of cytotoxic CD4 T cells for response to checkpoint blockade therapy

Various studies have suggested a critical role for cytolytic CD4 T cells in response to PD1/PDL1 based checkpoint blockade therapies. In bladder cancer, a cytotoxic CD4 T cell gene signature strongly predicts response to atezolizumab (anti-PDL1) (131). The cytotoxic CD4 T cells from the bladder cancer patients directly killed matched tumor blasts in an MHCII-dependent manner. Furthermore, the association between cytotoxic CD4 T cells and response to anti-PDL1 therapy only held in patients with immune-inflamed tumors, not in immune excluded or desert tumors where the CD4 T cells would not be in contact with the tumor cells (131). These two observations suggest that direct killing of tumor cells by cytotoxic CD4 T cells was important for the efficacy of anti-PDL1 in bladder cancer. Similar observations have also been made in Hodgkin’s lymphoma and colorectal cancer in the context of PD1 blockade (98, 132, 133). Those studies suggest that cytotoxic CD4 T cells are important for killing tumor cells resistant to killing by CD8 T cells. However, the relative importance of helper vs cytotoxic functions of CD4 T cells in those contexts is still unclear. We addressed this question using a murine leukemia model, where we showed that leukemia elicited CD4 T cells with both a Th1-like helper and a cytotoxic phenotype (92). A combination of a targeted therapy and PDL1 blockade elicited clonal expansion of leukemia-specific helper-cytotoxic CD4 T cells, which resulted in a significant survival benefit relative to treatment with the targeted therapy or PDL1 blockade alone. Interestingly, the combination therapy had minimal impact on the expression of cytotoxic molecules in the leukemia specific CD4 T cells. Instead, molecules associated with improved helper function and immune cell recruitment such as CCL4 and CD40L were significantly increased in the therapy-expanded CD4 T cells, which was associated with an improved expansion of GZMB+ CD8 T cells. Depletion of CD4 and CD8 T cells also resulted in a loss of treatment efficacy, suggesting that cytotoxic CD8 T cells expanded with signals from helper-cytotoxic CD4 T cells were required for the treatment efficacy. However, CD4 T cell depletion resulted in unform lethality while CD8 T cell depletion had a less penetrant but still significant impact on survival. This suggested that although the helper function of CD4 T cells was the major driver of anti-leukemia immunity, the cytotoxic function of the CD4 T cells likely still played an important role in leukemia clearance (92).

Interestingly in colorectal cancer, cytotoxic CD4 T cells with a Tr1-like phenotype are positively correlated with response to PD1 blockade, which was in direct contrast to their negative association with survival in the absence of PD1 blockade (98). The Tr1-like cytotoxic CD4 T cells in colorectal cancer were suggested to differentiate from Th1-like cytotoxic CD4 T cells in the tumor (98). Thus, we hypothesize that the anti-tumor role of cytotoxic Tr1 cells in this context is driven by the ability of PD1 blockade to preferentially enhance production of Th1 cytokines such as IFNγ over IL10 in Tr1-like CD4 T cells from human cancer samples (134). We hypothesize that this change in the ratio of IFNγ to IL10 in the cytotoxic Tr1-like cells plays a key role in their anti-tumor role in the presence of PD1 blockade (see **Figure 2**). IL10 is known to drive macrophage polarization towards a pro-tumor regulatory M2-like phenotype while IFNγ is known to drive macrophage polarization towards a stimulatory anti-tumor M1-like phenotype (135). Furthermore, IL10 inhibits the maturation of dendric cells (136) while IFNγ promotes the expression of MHCII on dendritic cells (137). In limited quantities, IL10 has beneficial anti-tumor effects by promoting the maintenance of stem-like CD8 T cells (138). PD1 blockade has been shown to recruit stem-like CD8 T cells to tumors and draining lymph-nodes, where their maintenance is essential for durable anti-tumor immune responses (139–142). Thus, the cytotoxic CD4 T cells in colorectal cancer are skewed towards a Tr1-like suppressive phenotype in the absence of treatment, while PD1 blockade can convert the pro-tumor Tr1-like cytotoxic CD4 T cells towards a more anti-tumor Th1-like phenotype. The ratio of IL10 to IFNγ produced by the Tr1-like cytotoxic CD4 T cells could be a key determinant in whether Tr1-like cytotoxic CD4 T cells have a net pro-tumor or anti-tumor impact (summarized in **Figure 2**).

### Cytotoxic CD4+ CAR T cells have improved persistence in cancer

Cytotoxic CD4 T cells have also shown to play key roles in long-term protection in the context of CAR T cell therapy. While CD8+ CAR T cells are highly efficient at killing target cells, they are more prone to activation-induced cell death that impacts their persistence. In contrast, CD4+ CAR T cells are less efficient at killing target cells but are less prone to activation-induced cell death (143). Indeed, CD4+ CAR T cells are shown to be more effective at persisting and in mediating protection against liquid and solid tumors *in-vivo*, in part due to their greater resistance to exhaustion vis-à-vis CD8+ CAR T cells (144, 145). Recent data from June and colleagues showed that CD4+ CAR T cells with a Tr1-like phenotype could be beneficial for long-term protection *in-vivo* (26). The authors tracked the proportion of CD4+ and CD8+ CAR T cells over time in patients with CLL. Although CD8+ CAR T cells dominated early on, the CD4+ CAR T cells eventually took over and were associated with long-term tumor control (26). A significant proportion of the CD4+ CAR T cells in the long-term survivors had a Tr1-like phenotype, including expression of IL10, EOMES and granzyme K (26). This suggests that a Tr1 phenotype is beneficial for persistence and long-term protection in the context of CAR T cell therapy. While CD4+ CAR T cells have been shown to persist longer, they have also been shown to induce a stronger cytokine release syndrome (146). Thus, the therapeutic utility of Tr1-like CD4+ CAR T cells in balancing tumor killing with mitigation of cytokine release syndrome would be worth exploring.

## Conclusions and future directions

Cytotoxic potential, as implied by the expression of granzymes, perforin, FasL, and/or granulysin, manifests in alpha/beta CD4+ T-cells in a variety of anatomical sites and inflammatory states. Cytotoxic potential has been observed in all known CD4+ memory/effector subsets, including FOXP3+ T-regulatory cells, Tr1s, Th1, Th2, Th17, and among non-classical subsets. Regardless of the specific CD4+ T-cell subset involved, cytotoxicity typically requires TCR signaling via recognition of cognate peptide-MHCII, context-specific inflammatory cues, and upregulation of transcriptional programs closely shared with CD8+ cytotoxic lymphocytes. While no master transcription factor is singly responsible for inducing cytotoxicity, key roles have been identified for EOMES, BLIMP1, HOBIT, and RUNX3. Particularly in the mucosal epithelium of the gut, cytotoxicity is linked with downregulation of the critical CD4-identifying factor THPOK, and upregulation of CD8αα. The breadth of observed cytotoxic CD4+ T-cell activity suggests that CD4s that mediate cell killing have unique functions from CD8+ T-cells, NK cells, or other cytotoxic cell types. Alternatively, cytotoxic potential may sometimes occur as a secondary consequence of stimulation, but not result in direct cell killing. Recognition of cognate peptides presented in an MHCII context is mediated principally by CD4+ T-cells and represents a setting where cytotoxic CD4+ T-cells would be expected to have a unique advantage. Confirming this, cytotoxic CD4+ T-cells are now known to directly target immune cells that constitutively express MHCII molecules. Of greater importance are observations that non-immune cells upregulate MHCII in inflammatory settings, resulting in CD4-mediated cell killing of epithelial and mesenchymal targets. These findings complement others showing that cytotoxic CD4+ T cells play important roles in infection, autoimmunity, and in both hematologic and solid malignancies.

Several outstanding questions remain in the field. First, how does cytotoxicity delivered by CD4+ T-cells affect downstream immunity? For instance, what is the impact of CD4 T cell-mediated killing of APCs? Secondly, what are the ontogenies and distinct functions of FOXP3^+^ and FOXP3^–^ regulatory T-cells that develop cytotoxic potential? Thirdly, what is the significance of cytotoxic CD4 T cells during ageing (147)? Is their increase simply a consequence of inflammaging; or do they have functional roles in controlling reactivated chronic viruses and/or removing senescent cells? Finally, what are the relative contributions of cytotoxic CD4s in anti-cancer immune therapies? The answers to these questions will illuminate the functional significance of this enigmatic CD4-T cell type.

## Author contributions

The manuscript was written by HV and ST. MF edited and revised the manuscript before submission.
